# Decreased gene expression from T7 promoters may be due to impaired production of active T7 RNA polymerase

**DOI:** 10.1186/1475-2859-4-3

**Published:** 2005-01-07

**Authors:** Joe GG Vethanayagam, Ann M Flower

**Affiliations:** 1 Department of Microbiology and Immunology, University of North Dakota School of Medicine and Health Sciences, Grand Forks, ND 58202-9037, USA; 2 Protein Expression Laboratory/NIAMS, National Institute of Health, Bethesda, MD 20892, USA

## Abstract

**Background:**

Protein expression vectors that utilize the bacteriophage T7 polymerase/promoter system are capable of very high levels of protein production. Frequently, however, expression from these vectors does not reliably achieve optimal levels of protein production. Strategies have been proposed previously that successfully maintain high expression levels, however we sought to determine the cause of induction failure.

**Results:**

We demonstrated that decreases in protein overproduction levels are not due to significant plasmid loss nor to mutations arising on the plasmid, but instead largely are attributable to chromosomal mutations that diminish the level of functional T7 RNA polymerase, resulting in decreased expression from the plasmid. Isolation of plasmid DNA from non-expressing strains and reintroduction of the plasmid into a T7 RNA polymerase-producing strain such as BL21(λDE3) reproducibly restored high level protein production.

**Conclusions:**

Our results suggest that a major contributing factor to decreased expression levels in T7 based systems is chromosomal mutation resulting in loss of functional T7 RNA polymerase. Consistent with this hypothesis, we found that optimal protein overproduction was obtained reproducibly from T7 promoters using freshly transformed cells that had not been subjected to outgrowth during which mutations could accumulate.

## Background

Often the first step in protein purification is production of the protein of interest in *Escherichia coli *using a gene expression system that allows selective overexpression of a cloned gene [1]. In the most desirable situation, expression of the cloned gene will consume the majority of the cellular resources, such that at the time of harvest the target protein makes up the bulk of the cell content. Complete purification of the protein then requires fewer selective extraction procedures and results in a more concentrated product. It is clear that optimizing the overexpression step is an effective method for maximizing and facilitating protein purification.

One commonly used system for achieving high levels of target protein production depends on the extremely selective nature of the bacteriophage T7 RNA polymerase for specific promoters [2-4]. In the pET system, the gene encoding T7 RNA polymerase is usually supplied by the host bacterial cell in the form of a λ lysogen which expresses the polymerase gene under control of the *lacUV5 *promoter [2,3]. The target protein is encoded on a plasmid in which the gene has been cloned so that transcription will be driven by the T7 RNA polymerase. Thus, expression of the T7 RNA polymerase gene is controlled by addition of IPTG to the growth media, and in turn, production of the polymerase controls expression of the plasmid-borne target gene.

The T7 RNA polymerase system is extremely effective, resulting in very high levels of target protein production, and the controllable promoters allow even the cloning and expression of genes encoding highly toxic proteins in *E. coli *[2,3]. However, anecdotal evidence has suggested that many investigators observe less than optimal expression from this system and even the lack of detectable protein overproduction. In our hands, the level of synthesis of proteins varied, not only with the specific protein encoded on the plasmid but also from sample to sample of the same protein.

Although poor protein synthesis, or complete lack of protein production, appears to be a common problem, strategies to cope with it are varied. Some protocols suggest "pre-induction" tests to determine specific colonies that will result in overproduction [5]. We found that this test, although simple, is tedious and can fail to identify expressor colonies. However, there are a number of effective strategies that have been proposed over the years [2,3,6-8]. While we support these recommendations, in this work we sought to determine the underlying cause of reduced expression from pET vectors in order to more effectively prevent problems with poor protein production levels.

## Results

We initially examined the conditions required for optimal target protein production from pET plasmids because we observed inconsistent results upon induction of plasmid-borne genes. This inconsistency was most apparent with the plasmid that encoded SecB, pJW25. Following the protocol provided [5], we streaked a frozen glycerol stock of BL21(λDE3) containing pJW25 for single colonies on LB plates with ampicillin, picked 4 colonies and grew small cultures to analyze overproduction of SecB. Only 1 of the 4 cultures demonstrated detectable overproduction of the SecB protein, and the level of overproduction from that one was not remarkable. Random selection of a large number of additional colonies (greater than 100 total) resulted in only 25–30% that synthesized levels of SecB that were clearly greater than the uninduced control. Further, if a culture that was determined initially to overproduce SecB was inoculated into a larger volume (500 ml), overproduction of SecB in the larger culture was not always detected. As we intended to grow strains for overproduction in a 5 liter fermentor, it was critical that the culture used reproducibly synthesize high levels of target protein.

**Figure 1:**
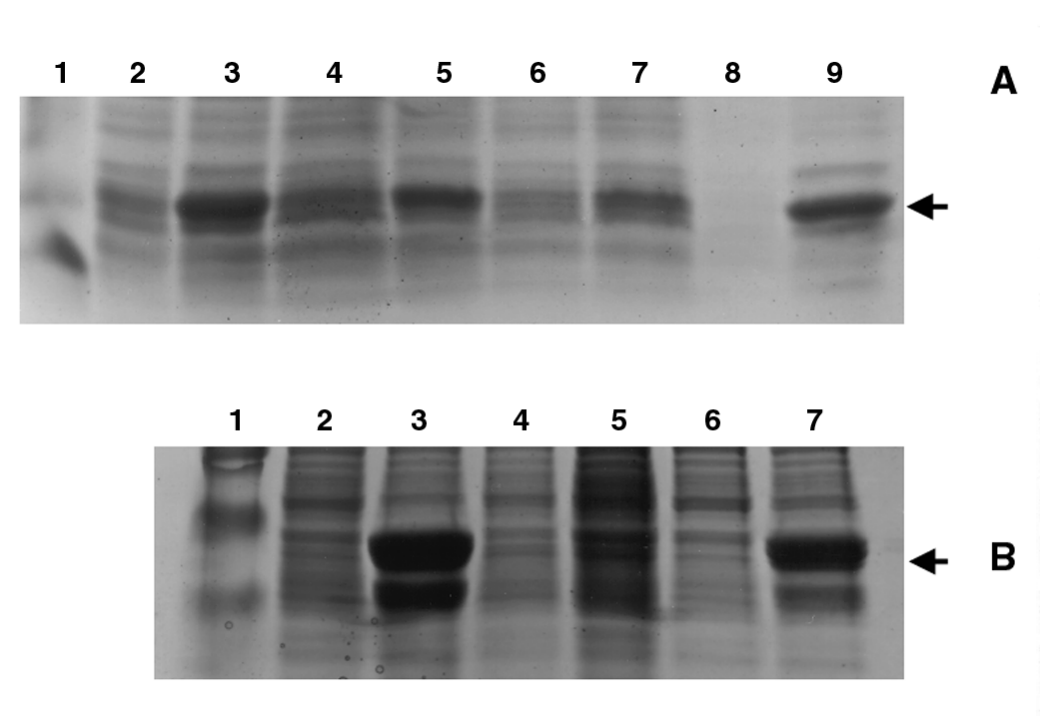
Protein production levels under various conditions. SDS PAGE shows protein levels of SecB (A) and proOmpA (B). Gels were stained with Coomassie Blue. A. 15% PAGE of cell lysates from BL21(λDE3) containing pJW25. Arrow indicates SecB migration. Lane 1, molecular weight marker; lanes 2 (uninduced) and 3 (induced) are samples from cells immediately after transformation; lanes 4 (uninduced) and 5 (induced) are the same strain after 3 subculturings; lanes 6 (uninduced) and 7 (induced) after 8 subculturings; lanes 8 (uninduced) and 9 (induced) are BL21(λDE3) retransformed with the plasmid rescued from the strain in lane 6. We note that lane 8 was unintentionally underloaded. B. 10% PAGE of cell lysates from BL21(λDE3) containing pJGGV. Arrow indicates proOmpA migration. Lane 1, molecular weight marker; lanes 2 (uninduced) and 3 (induced) are samples from cells immediately after transformation; lanes 4 (uninduced) and 5 (induced) are the same strain after 3 subculturings; lanes 6 (uninduced) and 7 (induced) are BL21(λDE3) retransformed with the plasmid rescued from the strain in lane 4.

While overproduction of SecB was unreliable from frozen stocks, we found that BL21(λDE3) that had been newly transformed with pJW25 overproduced SecB to a much higher level and that every colony examined synthesized SecB to a similar extent. Continued subculturing, however, resulted in a decrease or loss of overproduction (Figure 1A). These results suggested that with time the bacteria were losing the ability to overproduce SecB.

To verify that our observations were not unique to SecB, we examined a variety of other plasmids for overproduction of *E. coli *proteins in BL21(λDE3); the plasmid pJGGV encodes the secretory protein, proOmpA, pT7SecA produces SecA, the translocation ATPase [9], and pET11Tus encodes the Tus DNA binding protein [10]. Similar results were obtained with each of these plasmids. In all cases, newly transformed BL21(λDE3) gave rise to high levels of target protein production, while repeated subculturing decreased the level of expression, eventually to undetectable levels. The initial level of synthesis varied for each protein as did the severity of the effect of subculturing on overproduction. In all cases, however, the degree of overproduction was optimal immediately after transformation. Results are shown for data obtained with the pJW25 (SecB) and pJGGV (proOmpA) plasmids (Figure 1).

To more accurately determine the effect of subculturing on target protein production, protein levels were monitored as the strains were allowed to grow. Newly transformed BL21(λDE3) were grown as overnight cultures, then repeatedly subcultured as described in Methods. After each subculturing, when cultures reached early log phase (A_600 _= 0.4), a sample was removed and tested for the ability to overproduce plasmid-encoded protein. The amount of SecB synthesized by pJW25 decreased gradually with each subculturing, so that by the eighth subculture SecB protein production was minimal (Figure 1A). At this point, four of eight cultures no longer made detectable levels of SecB; the other four were greatly reduced in *secB *expression (data not shown).

The synthesis of proOmpA was even more dramatically affected by subculturing. Induction was abolished very quickly as there was no detectable overproduction in 15 out of 16 cultures after 3 subculturings (Figure 1B). SecA retained reasonable, but low, protein production levels through 11 subculturings, while Tus synthesis was nearly abolished after only 3 subculturings (data not shown). In all cases, cultures that continued to overproduce protein did so at greatly reduced levels. These results indicated that some proteins are more deleterious to the cell and that continued growth, even without induction, results in alterations to the strain such that overproduction is no longer possible.

We considered the following possible explanations for decreased synthesis of target protein with time: 1) the plasmid was not stably maintained in all bacteria, even with the constant presence of selective agent, 2) the plasmid was accumulating mutations that decreased expression levels, or 3) the bacterial strain accumulated mutations that inhibited production of functional T7 RNA polymerase, resulting in decreased or no expression from the plasmid. We examined each of these possibilities.

Previous protocols highly recommend that strains be examined for loss of plasmid prior to induction, and suggest that this is the major contributor to decreased expression levels [2,3]. However, our strains were grown with constant selective pressure for plasmid maintainence and subculturings were always made at high dilutions (1:1000) to minimize transfer of any β-lactamase from lysed cells to the fresh culture. Therefore, we felt that, while plasmid loss may be occurring, it probably would not be sufficient to account for the dramatic decreases observed in protein levels. To address this point, we prepared plasmid DNA from strains that had been newly transformed and overproduced proteins at high levels and also from the same strains that had been subcultured repeatedly and no longer synthesized high levels of target protein. We found that the amount of DNA present as observed by agarose gel electrophoresis was quite similar in both strains, regardless of the plasmid examined (data not shown). While the results we obtained are not quantitative, they indicated that plasmid loss alone could not account for the reduced level of expression.

To address possible plasmid loss more directly, we compared newly transformed strains and ones that had been subcultured repeatedly for their ability to form individual colonies on LB agar or LB agar containing ampicillin. As β-lactamase is a periplasmic enzyme, it is possible that sufficient leakage of the enzyme occurred to inactivate the ampicillin in liquid culture and allow growth of bacteria that no longer retained plasmid DNA despite the high dilution (1:1000) upon subculturing. If that were the case, we would expect the strain that had been subcultured many times to contain fewer plasmid-containing cells and therefore to form fewer colonies on LB+ampicillin. If no significant plasmid loss were occurring, we would expect the number of colonies to be equal whether or not ampicillin was present. We found that the pJW25-containing strain formed equivalent numbers of colonies on LB and on LB-ampicillin, even after subculturing eight times to result in a culture that did not overproduce SecB (data not shown). Thus, plasmid loss is not sufficient to account for the observed decrease in protein overproduction in these strains.

The second hypothesis was that the plasmids had suffered mutations that rendered them unable to overexpress target genes, for example mutations to the T7 RNA polymerase binding site or to the Shine Dalgarno region. To observe such a great decrease in protein production, a mutated plasmid must confer a growth advantage that results in cells containing the mutated plasmid to overtake the culture. Therefore, strains that no longer overproduce significant amounts of target protein should contain mutated plasmid. To test this possibility, we isolated plasmid DNA from strains that no longer synthesized significant amounts of target protein and retransformed BL21(λDE3) with the plasmid DNAs. If plasmid mutations were occurring, we would expect a large number of the transformants to be poor expressors. On the other hand, if plasmid mutations were not responsible for decreased expression, then we would predict that the new transformants would produce high levels of protein. Indeed, this was the result we observed. Every colony examined, from every plasmid tested, synthesized target protein efficiently after retransformation. Results with pJW25 and pJGGV are shown in Figure 1. The levels of protein production in newly transformed cells were similar to that of the original strain. This result indicated that plasmid mutation was not a significant cause of decreased target protein production from these plasmids.

**Figure 2:**
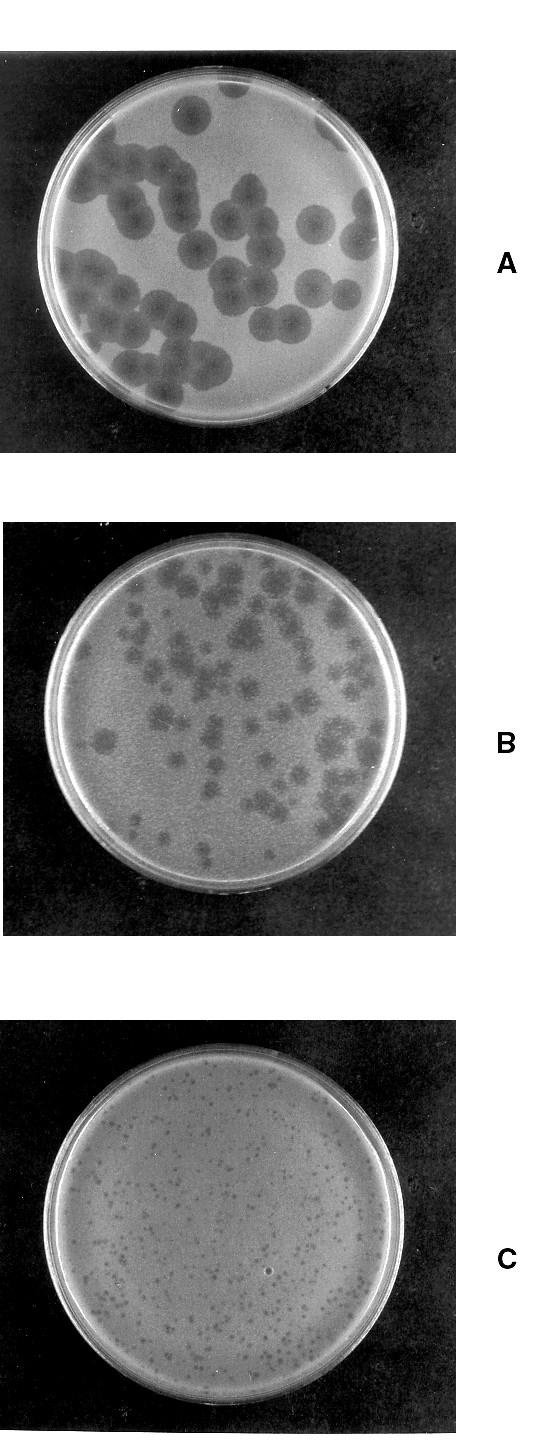
Growth of T7 phage Δ4107. A. Host strain was BL21(λDE3), 0.1 ml of phage from a 10^-8 ^dilution was plated. B. Host strain was BL21(λDE3) freshly transformed with pJW25. 0.1 ml of phage from a 10^-7 ^dilution was plated. C. Host strain was BL21(λDE3) carrying pJW25 after 10 subculturings. 0.1 ml of phage from a 10^-6 ^dilution was plated.

The third hypothesis was that mutation of the bacterial chromosome might lead to decreased production of active T7 RNA polymerase, in turn decreasing expression from the plasmid. We felt this explanation was probable as a single chromosomal mutation that diminished T7 RNA polymerase expression could be sufficient to abolish protein production from all resident plasmids and this mutant could quite likely overtake the culture in a fairly short period of time, even in the presence of antibiotic to maintain the plasmid. We examined this possibility by titering a mutant T7 phage, Δ4107, that requires host production of T7 RNA polymerase for growth [2]. T7 Δ4107 produced very large, clear plaques when grown on BL21(λDE3) (Figure 2A). When the host strain was BL21(λDE3) that had been newly transformed with pJW25, the titer decreased to about 25% the original, and the plaques produced were slightly smaller (Figure 2B). Furthermore, resistant colonies could be seen emerging within the plaques. After BL21(λDE3) containing pJW25 had been subcultured 10 times so that SecB was no longer detectably overproduced, the phage was again titered. The titer was about 25% of the original, but the plaques produced on this strain were very small and cloudy (Figure 2C). These results are consistent with loss of functional T7 RNA polymerase. We would not predict complete loss of plaque forming ability as the strain culture probably consists of a mixed population at the time of plating; that is, while most cells in the population would be derived from a mutant in which there is little or no functional T7 RNA polymerase, there may be some cells that have not suffered a mutation and would therefore support growth of the phage. We conclude therefore, that a major cause of decreased production of proteins from pET plasmids may be a decreased level of functional T7 RNA polymerase due to mutations in the BL21(λDE3) chromosome.

The loss of T7 RNA polymerase activity could be due either to mutation of the polymerase gene or excision of the λ prophage. To distinguish between these possibilities, we performed PCR using primers that amplify an 1100 base pair fragment of the T7 RNA polymerase gene. As template DNA, we used either BL21(λDE3), BL21(λDE3) freshly transformed with pJW25, or BL21(λDE3) containing pJW25 that had been subcultured eight times and no longer produced significant amounts of SecB. In all cases, a DNA product of the correct size was observed, indicating that prophage excision was not the major cause for decreased T7 RNA polymerase activity (data not shown). Therefore, our results suggest that mutation to the prophage, resulting in decreased levels of functional T7 RNA polymerase, was the predominant contributory factor for decreased production of target proteins.

## Discussion

Optimal overproduction is an important first step towards high quantity purification of target proteins. It is critical that one be certain that the culture used will synthesize large quantities of the protein of interest, particularly if it will be used for large scale production procedures, such as growth in a fermentor. When used correctly, the T7 RNA polymerase/promoter system is an excellent choice for such overproduction. However, care must be taken to achieve optimal protein production levels.

It had been suggested previously that plasmid loss is the primary cause for decreased expression from target genes in the pET system [2,3]. Our results differed. While plasmid loss may have occurred to a small extent, in the cases analyzed here the principal reason that lowered levels of protein production occurred was that mutations within the lysogen on the host bacterial chromosome resulted in decreased levels of functional T7 RNA polymerase. The fact that strains which no longer produced plasmid-encoded proteins also no longer supported vigorous growth of the mutant T7 phage, Δ4107, indicates that T7 RNA polymerase function was absent or greatly reduced in the majority of the population. Further, PCR amplification of the T7 RNA polymerase gene suggested that the reduction in polymerase function was due to mutation rather than to prophage excision.

Surprisingly, there was no clear growth disadvantage to strains carrying pJW25 as assessed by monitoring growth over time (data not shown). We do not think that this finding negates our proposal that chromosomal mutation occurs, however. Rather, this may explain why we see loss of expression more rapidly with some plasmids than with others; for example, synthesis of proOmpA was drastically decreased after only three subculturings. Apparently, even in the uninduced state, sufficient expression of target protein occurred, so that a detrimental effect resulted. If that target protein is sufficiently detrimental, selection for polymerase mutations will occur. For this reason, the steps that are taken to ensure optimal expression from target genes must be directed at limiting opportunities for mutation to the T7 RNA polymerase and for those mutants to overtake the bacterial culture.

Two methods have been suggested previously to ensure high levels of protein production; either screening individual colonies for protein synthesis [5] or testing the population of bacteria to assess the fraction of cells capable of overproduction [2,3]. The first is tedious and unreliable, while the second may be better used as an indicator of the degree of mutation that has occurred. However, as we found in this study, these precautions alone are not sufficient to ensure that the colony used for expression will be the highest protein producer possible. In particular, if any further growth of the strain occurs between the time of testing and the actual induction, mutations may occur that decrease expression levels. Our findings support previous recommendations for growth and storage of BL21(λDE3) containing pET plasmids [2,3,6,7] as the conditions described would limit mutation of the T7 RNA polymerase gene. Specifically, plasmid-containing BL21 (λDE3) grown to saturation in rich media will allow basal expression of the target gene [3]. Depending on the toxicity of the protein thus produced, these conditions will select for random mutations to the chromosomally encoded T7 RNA polymerase. Our studies also support the use of bacterial strains that contain the T7 lysozyme as the lysozyme is an inhibitor of the T7 RNA polymerase and will thus decrease uninduced expression even further [3].

We found that the quickest and most reliable method for obtaining optimal protein production was to freshly transform BL21(λDE3) with the desired plasmid, and use a colony directly from the transformation plate for expression studies with only a single subculture of an overnight culture. Should decreased levels of target protein production be observed, the problem can be simply remedied by isolating plasmid DNA from poor expressors and retransforming BL21(λDE3). However, we acknowledge that this approach may not be realistic for very large scale production facilities. Nevertheless, understanding the molecular basis for decreased protein production will facilitate development of techniques that will minimize conditions that may lead to selection and outgrowth of mutant strains.

## Conclusion

A common difficulty with overproduction of proteins using the T7 RNA polymerase based system is decreased target protein synthesis or even lack of detectable protein production. It is clear that synthesis of proteins, even at low basal levels, leads to the loss of induction capability. Effective strategies have been presented previously to avoid loss of expression, all of which are based on preventing basal expression levels. In this report, we demonstrate that a significant underlying mechanism leading to loss of expression is a decrease in functional T7 RNA polymerase, and selection of mutants unable to express the recombinant gene.

## Materials and methods

### Bacterial strains and plasmids

*Escherichia coli *B strain BL21(λDE3) was used for overproduction of proteins from plasmids containing T7 promoters and was obtained from Stratagene. All plasmids are derivatives of pET11 (Stratagene). Plasmids encoding SecB (pJW25) [5], SecA (pT7SecA) [9], and Tus (pET11Tus) [10] were generous gifts from Linda Randall, Bill Wickner, and Thomas Hill, respectively. The plasmid encoding proOmpA (pJGGV) was constructed in this laboratory by PCR amplification of the *ompA *gene from *E. coli *K12 strain MC4100 [11] using primers ompA-1 (gacctacccgggcatatgaaaaagacagctatcgc) and ompA-2 (ggtcatcccgggtgatcattaagcctgcggctgagttac, underlining indicates regions of homology to the *ompA *gene). The PCR product was digested with restriction enzymes NdeI and BclI and cloned into pET11c that had been digested with NdeI and BamHI. Standard protocols were used for PCR, restriction digests, ligations, and transformations [12,13]. Plasmid DNA was recovered from strains using a QiaPrep Spin Miniprep kit (Qiagen) following manufacturer's instructions.

### Induction and analysis of protein production

All strains were grown in LB medium [14]. When plasmid was present, ampicillin was added to a concentration of 100 μg/ml. Cultures were induced for protein production at an A_600 _of 0.4 by addition of IPTG to a final concentration of 1 mM. Growth was allowed to continue for 2 hours after addition of IPTG. Uninduced controls were grown the same except no IPTG was added. Cells were lysed by boiling in SDS [14], and proteins were analyzed by SDS polyacrylamide gel electrophoresis [13].

For experiments using newly transformed BL21(λDE3), colonies were picked directly from the transformation plate and inoculated into 5 ml LB containing ampicillin for overnight growth. The overnight culture was diluted 1:1000 into fresh LB with ampicillin and grown to an A_600 _of 0.4 for induction. Thus, the bacteria were subcultured only once. For continuous subculturing experiments, samples were removed before addition of IPTG and used to inoculate fresh LB plus ampicillin media at a dilution of 1:1000.

### T7 phage titering

Bacteriophage T7 mutant Δ4107 [2], which was a generous gift from Dr. William Studier, was grown for single plaques on BL21(λDE3) containing various plasmids using standard phage protocols [14].

### PCR amplification of T7 RNA polymerase

Genomic DNA was isolated using the Qiagen DNeasy kit. Oligonucleotides used as primers (t7pol1 – gattaacatcgctaagaacg and t7pol2 – gattcatgtcgatgtcttcc) were obtained from Midland Certified Reagents, Midland, TX. PCR was performed using the FailSafe PCR kit (Epicentre Technologies, Madison, WI) following manufacturer's recommendations. PCR products were visualized by agarose gel electrophoresis.

## Abbreviations used

IPTG – isopropyl-β-D-galactoside

PCR – polymerase chain reaction

LB – Luria-Bertani

SDS – sodium dodecyl sulfate

## Authors' contributions

JGGV was responsible for the original observations of inconsistent expression levels and the data for Figure 1, as well as the agarose gel analysis not shown. AMF performed the experiments for Figure 2 and prepared the manuscript. Both authors read and approved the final manuscript.

## References

[B1] Das A, Deutscher MP (1990). Overproduction of proteins in Escherichia coli:  vectors, hosts, and strategies. Guide to Protein Purification.

[B2] Studier FW, Moffatt BA (1986). Use of bacteriophage T7 RNA polymerase to direct selective high-level expression of cloned genes. J Mol Biol.

[B3] Studier FW, Rosenberg AH, Dunn JJ, Dubendorff JW (1990). Use of T7 RNA polymerase to direct expression of cloned genes. Meth Enzymol.

[B4] Tabor S, Richardson CC (1985). A bacteriophage T7 RNA polymerase/promoter system for controlled exclusive expression of specific genes. Proc Natl Acad Sci USA.

[B5] Randall LL, Topping TB, Smith VF, Diamond DL, Hardy SJS (1998). SecB:  a chaperone from Escherichia coli. Meth Enzymol.

[B6] Grossman TH, Kawasaki ES, Punreddy SR, Osburne MS (1998). Spontaneous cAMP-dependent derepression of gene expression in stationary phase plays a role in recombinant expression instability. Gene.

[B7] Kelley KC, Huestis KJ, Austen DA, Sanderson CT, Donoghue MA, Stickel SK, Kawasaki ES, Osburne MS (1995). Regulation of sCD4-183 gene expression from phage-T7-based vectors in Escherichia coli. Gene.

[B8] Kuderova A, Nanak E, Truksa M, Brzobohaty B (1999). Use of rifampicin in T7 RNA polymerase-driven expression of a plant enzyme:  rifampicin improves yield and assembly. Protein Expr Purif.

[B9] Cunningham K, Lill R, Crooke E, Rice M, Moore K, Wickner W, Oliver D (1989). SecA protein, a peripheral protein of the Escherichia coli plasma membrane, is essential for the functional binding and translocation of proOmpA. EMBO J.

[B10] Hill TM, Marians KJ (1990). Escherichia coli Tus protein acts to arrest the progression of DNA replication forks in vitro. Proc Natl Acad Sci USA.

[B11] Casadaban MJ (1976). Transposition and fusion of the lac genes to selected promoters in Escherichia coli using bacteriophage lambda and mu. J Mol Biol.

[B12] Sambrook J, Fritsch EF, Maniatis T (1989). Molecular Cloning A Laboratory Manual.

[B13] Ausubel FM, Brent R, Kingston RE, Moore DD, Seidman JG, Smith JA, Struhl K (1994). Current Protocols in Molecular Biology.

[B14] Silhavy TJ, Berman ML, Enquist LW (1984). Experiments with gene fusions..

